# Identification of genetic contribution to ischemic stroke by screening of single nucleotide polymorphisms in stroke patients by using a case control study design

**DOI:** 10.1186/1471-2377-13-136

**Published:** 2013-10-03

**Authors:** Amit Kumar, Ram Sagar, Pradeep Kumar, Jitendra K Sahu, Ashoo Grover, Achal K Srivastava, S Vivekanandhan, Kameshwar Prasad

**Affiliations:** 1 Department of Neurology, Room No. 704, Neurosciences Centre, All India Institute of Medical Sciences, Ansari Nagar, New Delhi, India; 2 Division of Pediatric Neurology, Department of Pediatrics, PGIMER, Chandigarh, India; 3 Indian Council of Medical Research, New Delhi, India; 4 Department of Neurobiochemistry, All India Institute of Medical Sciences, New Delhi, India

## Abstract

**Background:**

Stroke is the second most common cause of death and disability worldwide. It is a multi-factorial disease influenced by both environmental and genetic factors. Studies from the different ethnic regions of world have reported variable results on association of Apolioprotein E (APOE), Methylenetetrahydrofolate reductase (MTHFR), Endothelial Nitric Oxide Synthase (ENOS), Factor V Leiden (F5), Cytochrome P450 4F2 (CYP4F2), beta-fibrinogen and Phosphodiesterase 4D (PDE4D) gene in stroke. There has been substantial evidence from the European descent genetic studies showing that genetic risk of stroke varies as per specific subtypes of ischemic stroke.

This study aims to test the hypothesis that above mentioned encoding gene polymorphisms are associated with stroke and to determine whether risk varies as per specific subtypes of stroke.

**Methods/Design:**

The study design would be case–control study. Six hundred cases with diagnosis of stroke and 600 age and sex matched controls will be recruited. Controls will be matched in 1:1 ratio. Baseline and demographic data will be collected in standardized data collection form. Four ml of blood will be collected in EDTA coated vial and will be used for DNA isolation. Genotyping will be done by using PCR-RFLP method. For the reconfirmation of RFLP results, PCR product of each genotype in triplet for all the selected polymorphism will be sent for DNA sequencing. Data will be analyzed using conditional logistic regression to determine odds ratio associated with the above genes.

**Discussion:**

This protocol will assess the association of above mentioned gene polymorphisms with ischemic stroke in North Indian Population. This study will also helpful to determine genetic component of stroke and whether variation in genetic risk as per different subtypes of stroke.

## Background

Stroke has emerged as the second commonest cause of mortality worldwide and is a major public health problem. Stroke has accounted for nearly 5.7 million deaths worldwide in 2005 [1]. More than two-thirds of these deaths occur in less developed countries [2]. The latest available estimates from Indian Council of Medical Research (ICMR) indicate that in 2004, 41% deaths and 72% disability adjusted life years (DALY) among non-communicable diseases were attributable to stroke [3]. Incidence of stroke is rapidly increasing in low and middle income countries. Incidence of stroke in South Asian countries have increased by more than 100% while this is deceased by 42% in developed European countries in last four decade [4]. WHO estimates suggest that by 2050, 80 percent of stroke cases in the world would occur in low and middle income countries, mainly India and China [5].

Stroke is a multi-factorial polygenic, complex disease resulting from combination of vascular, environmental and genetic factors [6]. There is large body of evidence, suggesting a genetic component to stroke. Animal model studies, twin and family-based association studies have suggested the substantial genetic component of stroke [7]. There is nearly several fold increase in the prevalence of stroke among the monozygotic compared with the dizygotic twin pairs suggest that substantial contribution of genetic in the risk of stroke [8].

Identification and management of new risk factors to improve prevention remains an important strategy to reduce the human and economic burden of stroke [9]. Currently, there are only few drugs available for the stroke treatment. Therefore, there is a clear need to identify new drug targets. In view of ongoing advances in personalized medicine on the basis of individual genetic makeup, identification of SNPs for stroke will be helpful for the development of specific drug target and patient stratification for the treatment of stroke according to individual genetic make-up. There is wide variation in study results of various published candidate gene studies across the different part of globe. Frequency of SNPs variant varies across and within ethnic groups due to complex environment gene interaction. Factors responsible for varying study results include different study design, variation in sample size, and inadequate characterisation of phenotypes and lack of case control matching. There is a clear need to conduct more studies meeting the requirement of standard guidelines such as STEREGA for genetics study. We planned this study following this guideline. The study will add to existing small body of evidence on genetics of stroke in India. In this proposal, we plan to study some important SNPs (Table 1) in North Indian stroke patients by using candidate gene approach. We will also evaluate if genetic risk varies across subtypes of ischemic stroke according to TOAST classification [10]. We will also examine whether single or in combination of genotypes can predict the clinical outcome six months after stroke. The objectives of the present study are (i) to determine association of putative risk factor gene polymorphisms (Table 1) and their haplotypes in ischemic stroke with different subtypes of ischemic stroke in North Indian population, (ii) to investigate whether any association found between ischemic stroke and the panel of tested polymorphisms is influenced by sex, age, or smoking status and other environmental risk factors due to gene-environment interactions, and (iii) to determine the frequency distribution of the above mentioned polymorphisms will be accordance with Hardy Weinberg equilibrium.

**Table 1:** Location and characterization of polymorphisms to be studied

**S. No.**	**Gene**	**Polymorphism**	**Role in mechanism of stroke**
1.	APOE	Epsilon 2/3, epsilon 3/3, or epsilon 3/4	Arthrosclerosis
2.	MTHFR	C677T	Arthrosclerosis, venous thrombosis
3.	ENOS	G894T	Arthrosclerosis
4.	Factor V Leiden	1691G>A (Arg506Gln)	Venous thromboembolism
5.	CYP4F2	1347 G/A	Vasoconstriction, increase in vascular tone
6.	beta-fibrinogen	(C_148_→T)	Carotid arthrosclerosis
7.	Phosphodiesterase 4D (PDE4D)	SNPs 45 (rs12188950), SNP 83 (rs 966221) and SNP 87 (rs 2910829),	Arthrosclerosis

### Justification for selection of genes

#### Methylenetetrahydrofolate reductase (MTHFR)

Several case control and prospective studies demonstrated that moderate elevation of plasma homocysteine (Hcy) is a potential risk factor for cardiovascular disease, venous and arterial thrombosis including stroke [11]. Methylenetetrahydrofolate reductase (MTHFR) is an important enzyme in the metabolism of homocysteine. A C677T mutation in this enzyme leads to a reduction in enzyme activity and an elevation of plasma Hcy. Some studies reported that C677T mutation is associated with Ischemic stroke but others failed to find association. A meta-analysis with 22 studies published in 2004 showed odds ratio (OR) 1.24 (CI 1.08 to 1.42) having CC genotype and when compared with TT genotype. A meta-analysis of 15 case control studies included 2034 cases and 4485 controls showed significant associations between the MTHFR C677T genetic polymorphism and risk of hemorrhagic stroke under dominant model (OR, 1.61; 95% CI, 1.3 to 1.9) and in recessive model (OR, 1.6, 95% CI, 1.4 to 2.0) [12]. A meta-analysis showed significant association between elevated plasma homocysteine levels and TT genotypes of MTHFR C677T polymorphism in healthy South Asians [13].

#### Apolipoprotein E (APOE)

Apo-E protein contributes a major role in lipid transport and metabolism and is also significantly expressed in brain. Apo E is one of the commonly studied genes in vascular and neurodegenerative diseases. Its protein product are composed of glycoprotein with 3 common isoforms, E2, E3, and E4, encoded by the respective alleles ε2, ε3, and ε4, giving rise to 6 genotypes. There is substantial evidence of association of Apo ε4 allele with elevated LDL cholesterol levels and thereby increases the risk of cardiovascular diseases. It has been shown that elevated level of Apo E in plasma is an important risk factor for stroke. Apo E polymorphism can also modify the risk of other modifiable risk factor e.g. the effect of cigarette smoking on ischemic stroke may be higher in young adults who carry the variant apo e 4 allele [14]. A meta-analysis published in 2006 [15] which included 4096 cases and 16117 controls suggested Apo E contribute the risk of stroke with an OR, 1.11; 95% Cl, I.01 to 1.22).

#### Endothelial nitric oxide synthase (eNOS)

The eNOS gene is located on chromosome 7 (7q35-q36) and consists of 26 exons. It codes for an enzyme that generates Nitric Oxide (NO) in the vascular endothelium. NO mediates the vasodialation in the endothelium and it also inhibits the adhesion of platelets and leukocytes and limits the oxidation of atherogenic low-density lipoproteins in the vascular endothelium. Impaired endothelium-dependent vasodilatation is a common feature of atherosclerotic vessels, which seems to be partly due to the reduction in the activity of vascular endothelial nitric oxide synthase. Impaired nitric oxide-dependent vasomotor reactivity has been implicated in the pathophysiology of stroke. Since it has an important role in the physiology of the vasculature, genetic variation could alter the expression and activity of eNOS, and therefore contribute to the development of stroke. A meta-analysis [16] published in 2009 suggested, TT genotype of G-894T polymorphism has no association with ischemic stroke (OR, 1.14; 95% CI, 0.99 to 1.31). A recent meta-analysis included 27 studies suggest the positive association between eNOS gene 4b/a, T-786C, G894T polymorphism and ischemic stroke [17].

#### Factor V Leiden

The factor V gene is located on chromosome 1.q23, spans more than 80 kb and contains 25 exons. In exon 10 where G nucleotide is replaced by A nucleotide results in an amino acid substitution of arginine at position 506 by glutamine. This substitution blocks a major cleavage site of activated protein C (APC), thereby resulting in a decreased ability of APC to inactivate the procoagulant factor Va which result in hypercoaguable state that leads to an increased risk for venous thromboembolism. A meta-analysis showed that Factor V Leiden is associated with ischemic stroke in young adults, particularly in patient populations where there is an increased clinical suspicion of prothrombotic state. A meta-analysis of 767 cases and 4020 controls observed that Factor V Leiden 1691 G→A is associated with patients with adult venous thrombosis patients (OR 2.40; 95% CI, 1.75 to 3.3) [18].

### beta-fibrinogen (−148 C/T) gene polymorphism

Plasma fibrinogen is an important component of the coagulation cascade, as well as important determinant of blood viscosity and blood flow. Increased level of fibrinogen may promote a prothrombotic or hypercoaguable state and may explain the involvement in risk of stroke. Fibrinogen is encoded by three separate genes located in a 50-Kb cluster on the long arm of chromosome number 4, which encode α, β and γ chains. The rate limiting step in fibrinogen formation is the synthesis of the β-polypeptide chain regulated by a β-fibrinogen promoter. C148T polymorphism of beta is located close to an interleukin-6 responsive element and may affect fibrinogen gene expression, mainly in response to acute phase reaction. Studies support C148T polymorphism is associated with increased plasma fibrinogen level in both men and women in general population. There are numerous studies describing an association between plasma fibrinogen levels and coronary heart disease and stroke and carotid atherosclerosis. Fibrinogen concentration is controlled by genetic and environmental factors, including smoking, obesity, use of contraceptives, trauma, and lack of exercise, which have been reported to elevate fibrinogen concentrations. Fibrinogen level also increases with age and in the presence of diabetes mellitus, hypertension, or lipid abnormalities. A meta-analysis of eleven studies in Chinese population included 1223 cases and 1433 controls showed the pooled OR of susceptibility to cerebral infarction for -148T allele carriers was 1.32; 95% CI, 1.12 to 1.55 when compared to wild homozygous [19].

#### Phosphodiesterase 4D (PDE4D)

PDE4D gene is located on short arm of chromosome number 5q12 and consists of 24 exons. The gene expresses nine different functional protein isoforms through alternative splicing or the use of differential promoters. The different PDE4D variants are expressed in various tissues including brain, lungs, kidneys, monocytes, B and T lymphocytes and vascular smooth muscles [20]. PDE4D is the family of enzyme which breaks phosphodiester bond of cAMP degrades them and maintains the appropriate level and duration of action of cAMP within the cell. Cyclic AMP is secondary signaling molecule which involves provoking genes to produce inflammatory mediators by several types of inflammatory cells and arthrosclerosis. PDE4D degrade cAMP, therefore responsible for subsiding inflammatory process by stop massaging through cAMP to genes that produce inflammatory proteins. Polymorphism in this gene may affect catalytic efficiency of PDE4D. In 2002, the deCODE group published the results of a genome wide screen for stroke susceptibility genes in Iceland [21]. Among 260 phosphodiesterase 4D (PDE4D) single-nucleotide polymorphisms (SNPs) examined, six were significantly associated with stroke after adjustment for multiple comparisons.

#### Cytochrome P450 4F2 (CYP4F2)

The cytochrome P450 4F2 (*CYP4F2*) gene, prominently expressed in human kidney and liver, encodes a ω-hydroxylase that catalyzes the metabolism of arachidonic acid, leukotriene B4, and tocopherol. The 20-hydroxyeicosatetraenoic acid (20-HETE), derived from arachidonic acid by CYP4F2 in the kidney, acts as a natriuretic and vasoactive eicosanoid and plays an important role in the control of renal function and systemic BP. Considerable evidence showed that altered renal 20-HETE content and *CYP* genes may play an important role in hypertension and ischemic stroke. One study reported from south India found association of 1347 G/A polymorphism (rs2108622) with stroke [22].

## Methods/Design

### Ethical considerations

Study Protocol has been approved from Institutional Ethics Committee *(Ref No: IEC/NP-122/2012 & RP-14/2012)*.

### Design of study

Case control study design.

### Patients and methods

Patients will be eligible if they meet all the inclusion criteria and none of the exclusion criteria.

### Selection of cases and controls

#### Inclusion and exclusion criteria for cases

Inclusion criteria for cases

a) Diagnosis of stroke as defined by World Health Organization, b). NCCT-Head consistent with ischemic stroke, c). Stroke onset within three years before the recruitment, d). Age 18–85 years (both sexes), e). Willingness to provide written informed consent by self or legal representative, f). Should be resident of North India (residing for last one year or longer), g). Be ’North Indian’. A North Indian defined in consultation with Department of Paediatric (Genetics) AIIMS and Genetics Department of the Institute of Genomics and Integrative Biology (IGIB) as having all of the following:

(i) The subject’s birth place should be in North India, (ii) Their ancestor (2 generation) should be North Indian, (iii) Subjects must know any North Indian language which include any language spoken in North India.

For this study, North India included the states of Himachal Pradesh, Bihar, Delhi, Punjab, Uttar Pradesh, Madhya Pradesh, Uttrakhand, Jharkhand, Rajasthan, Jammu and Kashmir and Haryana.

Exclusion criteria for cases

a). Stroke associated with pregnancy, b). Stroke associated with surgery, c). Unwillingness to provide written informed consent (by self or legal representative).

### Inclusion and exclusion criteria for controls

Inclusion criteria for controls

a). Age (−5 to + 5) and sex matched, b). Controls have not had prior stroke by questionnaire for Stroke-free Status (QVSS) [23], c). Spouse or friends but not a relative (by blood), d). Age 18–85 years (both sexes), e). Should be Resident of North India (residing for last one year or longer), f). Willingness to provide written informed consent by self or legal representative, g). No evidence of any serious brain disorders, h). Be ’North Indian’. (North Indian criteria is same as above).

Exclusion criteria for controls

a). Unwillingness to provide written informed consent (by self or legal representative), b). Pregnancy, c). Subjects with any serious brain disorder.

### Definition of variables

Definitions of variables were modified from the study [24] and are as follows: ***Hypertension*:** Subjects will be considered to have hypertension if they either have the diagnosis of hypertension or treated for hypertension before the stroke or reference date, In addition, if a control will have no recorded blood pressure before the reference date but diastolic pressure of 95 mm Hg or more or a systolic pressure of 160 mm Hg or more on two or more occasions during the study evaluation, he or she will be considered to have hypertension. **Diabetes:** if a subject will have the diagnosis documented by a physician on the medical record or if fasting blood sugar level will be >126 mg/dl, **Dyslipidemia:** if they either will have the diagnosis of dyslipidemia or treated for dyslipidemia. ***Angina pectoris*:** chest discomfort or pain that described as heavy, tight, constricting, crushing, pressing, or squeezing. ***Smoker*:** Person will be defined as regular smoker if a person smoking ≥1 cigarettes daily, Biris, Cigar for proceeding>3 months. ***Body Mass Index (BMI)*:** BMI will be calculated by weight in kilograms divided by the square of height in meters. ***Family history of Stroke*:** A positive family history of stroke will be considered if a subject’s first-degree relative (parent or sibling) had a stroke. ***Myocardial Infarction*:** The diagnosis will be based on clinical history of acute myocardial infarction; ***Migraine*:** subject will be considered to have a history of migraine if patients have a prescription for specific antimigraine therapy or diagnosis of migraine and a prescription for a potential antimigraine medication or analgesics in the absence of any other explanatory diagnosis within 1 years before the index date; ***Transient Ischemic Attack*:** TIA will be defined as subjects with focal neurologic symptoms relating to focal cerebral, brain stem, or retinal ischemia with abrupt onset and complete resolution within 24 hours. ***Economic status*:** the economic status of the subjects will be assessed based on the ownership of different commodities in house hold, mainly two wheeler, refrigerator, computer or car. The economic status will be classified into two classes: *Low* – not possessing any of the four, *High*: possessing either two- wheeler or refrigerator or computer or car. **Physical activity** will be determined on the basis of job profile of the subjects in which *Sedentary* (mostly sitting e.g. shopkeeper, clerk; *Moderate* physical activity (involves walking e.g. salesman, nurses, house work etc.); *Heavy physical work* (carrying, lifting e.g. labourer, coolie).

### Matching criteria for control to case

Matching is required for case control study for the elimination of bias in comparison between cases and controls. It assures that no large imbalance between cases and controls occurs. Controls will be matched with gender and age (± 5 years of cases) in 1:1 ratio. As Spouses would have similar environmental exposure as cases spouse can be used as control for case control stroke genetics study [25]. We will use spouses of cases as a match for other cases for age and sex matching. If there would be any lag and unavailability of spouse we will recruit age and sex match control from relatives/patients attending neurology department for treatment other than stroke and fulfilling the inclusion criteria for recruitment of controls.

### Stroke classification

We will use TOAST classification for the determination of stroke subtypes [10]. In TOAST classification stroke has five subtypes (i) Large vessel stroke (ii) Small vessel stroke (iii) Cardioembolic stroke (iv) Other determined aetiology (v) Undetermined aetiology.

### Sample size

Sample size calculation for all gene polymorphism was based on the parameters of our meta-analysis of association of MTHFR polymorphism with stroke. In this meta-analysis result prevalence for TT variant genotype were 0.17 and 0.13 in cases and control respectively, Odds ratio for this polymorphism was 1.31 Assuming 80% power and 5% alpha, with one control per case, we obtained estimated minimum sample size 578 cases and 578 controls. 600 cases and 600 controls will be included in this study to compensate for any loss of sample.

### Blood sample collection, processing, storage and genotyping

Four ml of blood sample will be collected in EDTA coated vial from all consenting participants in single-time venipuncture from antecubital vein. Samples will be used for Genomic DNA isolation from white blood cells by using phenol chloroform isolation method and extracted genomic DNA will be dissolved in 200-600 ml TE buffer depending upon the concentration of DNA and will be stored at -20°C. DNA will be isolated in weekly basis for isolation of good quality of DNA. Its quality will be checked first in 0.8% agarose gels. Quality of DNA in per μl will be checked in Nanodrop spectrophotometer. The purity of the DNA sample will be ascertained by calculating a 260/280 ratio. The ratio between 1.5-1.8 will be acceptable for PCR amplification. Genotyping will be done by the PCR – RFLP method. The PCR-RFLP results will be confirmed by direct sequencing of three samples of each genotype of all the chosen polymorphisms.

### Data collection and data analysis

Data will be recorded in standardized data collection forms. The data will be managed and analyzed using statistical software SPSS version 17. *T*-test will be used for continuous variables. Chi Square tests and logistic regression techniques will be used when outcome variable are categorical (Present/Absent). Association between each risk factor of interest and stroke will be performed using a conditional logistic regression approach. Odds ratio (ORs) and corresponding confidence intervals will be calculated for each polymorphism. A multivariable conditional logistic regression analysis will be performed for adjustment of other covariates. The other associated risk factors will be treated as covariates in examining the associations with stroke. Significance in the final model will be defined as P<0.05. Phenotype-genotype and genotype-environment interaction will be analyzed using routine statistical methods. Haplotypes will be constructed from selected SNPs and its association with stroke will be estimated using regression techniques

### Outcome measure

The primary outcome of the present study is to determine whether singly or in combination of any of selected polymorphisms are associated with stroke or its subtypes. Cases will be compared with controls to frequency and distribution of susceptible allele. Other outcome measure includes association of gene polymorphisms with different environmental exposures such as hypertension, smoking, diabetes, dyslipidemia etc. Data from association of genetic polymorphism with outcome of stroke is lacking. Determining the relationship of genetic variations with the stroke outcome will improve our understanding that how variations in the genes influence the stroke outcome. In present study we will assess the outcome of recruited patients at six months by telephone to assess the status of patients. One research worker will assess the Barthel Index and modified Ranking scale after the six months of stroke. Chronic stroke patient who will come after the six months of onset of stroke their six months Barthel index and modified Ranking scale will be assessed retrospectively.

## Discussion

Several candidate genes association studies with Apolioprotein E (APOE), MTHFR, ENOS, Factor V Leiden, cytochrome P450 4F2 (CYP4F2), beta-fibrinogen Gene and PDE4D gene polymorphism in stroke resulted in conflicting results. This study will have taken appropriate measure to deal with this issue.

### Strengths of the study

There are inconsistent results on candidate gene association studies in stroke and they have been criticized for non-replicability [26]. The possible reasons for the wide variations includes (i) variation in the methodology, (ii) lack of proper selection of the cases and control, (iii) lack of proper definitions of variables for phenotypic and genotypic data collection, (iv) insufficient sample size, (v) inappropriate control, who are often not screened, incorrectly matched with patients, and recruited in absence of strict criteria mainly from hospital staffs and known to researchers. Present study has taken measures to limit the above shortcomings.

Correct definitions of cases and of all the variables are crucial for a case control study. There is 39 fold increase in sample size for genetic association studies when the misclassification rate is 5% and disease prevalence is 1% [27]. There are two levels at which misclassification can occur: First, there could be misclassification of cases and controls. Second, there could be misclassification among cases (or controls) on whether they have the variable (risk factors) or not. A clear definition of cases and operational definition of variables helps to minimize the misclassifications. Misclassifications of the second kind may be differential or non-differential. While differential misclassification introduces systematic error in the measurement, the non-differential misclassifications introduce random error (Schlesselman, 1982) [28]. Many of reported candidate gene studies did not provide definition of cases and controls. Definitions of variables vary across the different scenario and even in the same disease at different time periods. For instance, in the acute phase of stroke, there is transient reactive hypertension. Therefore, the usual definition of hypertension systolic blood pressure 140 or above/ or diastolic blood pressure 90 or above may misclassify patients. Therefore, it is essential to provide operational definitions of variables in case control study. This study attempted to provide a clear definitions of cases and controls as well as clear operational definitions of variables.

### Selection of controls

Selection of controls for the genetic case control study is the most difficult part of study. Schleselman point out, (Schlesselman, 1982) “the control series is intended to provide an estimate of the exposure rate that would be expected to occur in the cases if there were no association between the study disease and exposure”. In present study, we will use age and sex matched controls. Controls will be selected from hospital mainly from spouses which serve as better controls as they would have similar environmental exposure [25]. Spouses of cases will be matched to other cases with same sex. This will allow us to reduce the bias due to effect of environment exposures between cases and controls. Any lag in case of unavailability of spouse will be fulfilled by age- and- sex match unrelated patients/relative (unrelated by blood) who will be seeking medical care from Neurology Outpatient Department for conditions other than stroke and fulfilling inclusion and exclusion criteria of the present study. This will allow us to select the controls to approximate distribution of exposure among them to that in the population from which cases arise. We attempted to properly define the geographical area from cases and controls will be recruited. This will allow us to maximize genetic homogeneity in the study population.

#### Bias in genotyping

Genotyping error may lead to misclassification of 30% of samples [27]. It is essential to reduce the genotyping error for genetic association studies to reduce the false positive or negative association of allele to the diseases. There are various reasons for genotyping error such as failure to identify the triallelic SNP, partial digestion of PCR product in PCR-RFLP method, lack of blinding to personnel undertaking genotyping for case control status of samples. In our study research personnel, who will be responsible for genotyping, will be blinded to case control status of the sample. In several circumstances there is incomplete digestion of PCR product which may lead to bias in the genotyping. To deal with this issue in our study, RFLP image will be read by the two investigators separately. A third person will resolve the issues wherever discrepancies occur.

### Hardy-Weinberg equilibrium

Original proportion of genotypes in the population remains constant from one generation to next in the absence of evolutionary forces. Many factors lead to deviation from the hardy Weinberg equilibrium like population stratification and random chance. It is essential to check in the genetics study whether distribution of observed alleles and genotypes are in accordance with Hardy Weinberg equilibrium in both cases and controls. Minor to modest deviation may provide the false positive association of variant with the disease. In several published candidate gene studies have not presented data whether their genotypic frequencies followed the Hardy-Weinberg equilibrium. In our study we will check whether the frequency distribution of genotypes following Hardy-Weinberg equilibrium or not. If there would be deviation from Hardy-Weinberg equilibrium, essential statistical measure will be adapted to deal with this issue.

### Outcome data

Recovery of stroke greatly varies from individual to individual after the onset of stroke depends upon clinical, radiological parameter and individual genetic composition. Association data of genotype with the better recover or poor recovery after the onset of stroke are lacking in the literature. In our present study we will examine association of specific genotype with six month stroke outcome.

We have taken appropriate measure to improve the quality of study. This study will provide quality data on association of above polymorphisms, in accordance with STEREGA guidelines. This study will improve our understanding of its risk factors and will facilitate identification of individuals at increased risk of disease. A clear and comprehensive understanding of genetic risk may promote advances in gene therapy and in the development of novel pharmaceutical agents for the treatment for stroke. Identification of genetic risk factors will be helpful for the better prevention of stroke.

## Competing interests

The authors declare that they have no competing interests.

## Authors’ contributions

AK is research postgraduate at All India Institute of Medical Sciences, New Delhi and has co-written the final protocol and responsible for the project management. RS and PK helped in developing the manuscript and responsible to data collection and genotyping. JKS and AG contributed to writing the portion of the manuscript. SV and AKS are co-investigators and contributed to developing and writing the final protocol. KP is the principal investigator of this project and has developed the protocol to the final version. All authors read and approved the final manuscript.

## Pre-publication history

The pre-publication history for this paper can be accessed here:

http://www.biomedcentral.com/1471-2377/13/136/prepub
